# Synergizing High-Quality Tourism Development and Digital Economy: A Coupling Coordination Analysis in Chinese Prefecture-Level Cities

**DOI:** 10.3390/e27101061

**Published:** 2025-10-12

**Authors:** Yuyan Luo, Yue Wang, Ziqi Pan, Huilin Li, Bin Lai, Yong Qin

**Affiliations:** College of Management Science, Chengdu University of Technology, Chengdu 610059, China

**Keywords:** high-quality tourism development, digital economy, coupling coordination, random forecast, prefecture-level city

## Abstract

The rapid development of the digital economy (DE) provides a new driving force for high-quality tourism development (HQTD). How to coordinate HQTD and DE is an urgent issue to be resolved. In this study, the coupling coordination degree (CCD) between HQTD and DE in Chinese prefecture-level cities is analysed using the CCD model, and the factors driving CCD are identified by Shapley additive explanations (SHAP). The results show that (1) Chinese city-level HQTD and DE show a rising trend from 2010 to 2019. The national average rises from 0.1807 and 0.2434 in 2010 to 0.2318 and 0.4113 in 2019, respectively, with HQTD’s development lagging noticeably behind DE. (2) CCD exhibits marked inter-regional disparities and intra-regional clustering. The northwest region has the lowest values, with many cities’ CCD below 0.5, indicating an imbalanced status. In 2019, all cities in the eastern region are in a balanced status, with Shanghai exceeding 0.8. (3) Total social retail sales per capita and percentage of tertiary sector are the key drivers of CCD; economic development and urbanisation rate exhibit a non-linear relationship with CCD. The CCD in developed cities in the east and north is driven by consumption, whereas the northwest region is primarily influenced by factors related to labour capital. Based on these conclusions, some policy implications are provided for the synergistic development of HQTD and DE.

## 1. Introduction

Tourism has long been recognised as a key driver of economic growth, enhancing the well-being of residents and fostering regional development. In 2023, the tourism sector contributed 4.24% to China’s GDP [1]. However, with the rapid growth of the Chinese tourism industry, the traditional model of quantity-driven development is no longer sufficient to meet its evolving needs. Several United Nations Sustainable Development Goals (SDGs), including SDG 11, SDG 13, SDG 14, and SDG 15, are closely linked to tourism development, particularly in the areas of ecology, urbanisation, and sustainability [2]. Consequently, there is a pressing need for the Chinese tourism industry to undergo transformation and upgrade to achieve high-quality development. As a highly information-intensive industry, tourism is increasingly shaped by the digital economy, which plays a critical role in fostering its high-quality growth. The rapid advancement and application of digital technologies offer both unprecedented opportunities and challenges to the sector. The digital economy has revitalised tourism, driving innovation, transformation [3], and improvements in service quality and efficiency [4]. Additionally, it has introduced new business models and development paradigms. In response, the Chinese Ministry of Culture and Tourism, together with the National Development and Reform Commission, issued a joint directive promoting the deep integration of “Internet + Tourism” by 2025, aiming to position the digital economy as a key driver of the tourism sector’s transformation and high-quality development. In this context, it is particularly crucial and urgent to examine the coupling and coordination between high-quality tourism development and the digital economy. Such an analysis can provide a reference for policymakers to promote the integration of high-quality development of tourism and the digital economy.

The relationship between the digital economy (DE) and tourism development has received considerable attention, particularly regarding the role of digital technologies in promoting tourism growth. However, tourism development and high-quality tourism development (HQTD) are often confused, and empirical research on the bidirectional interaction between DE and HQTD systems remains lacking, particularly concerning the interactive effects of HQTD and DE at higher spatial scales. Therefore, this study focuses on the HQTD, exploring the interactive bidirectional relationship between HQTD and DE, providing insights to inform policymaking and promoting the coordinated development of both systems. Taking prefecture-level cities in China as the research object, this study adopts the entropy weight method to construct a comprehensive evaluation system of HQTD and DE. Subsequently, the coupling coordination degree model (CCDM) is used to assess the level of coordinated development between HQTD and DE, and the spatial and temporal evolution trend of the coupled coordination degree (CCD) is analysed using kernel density. Finally, the non-linear impact of drivers on CCD is explored using SHAP and machine learning.

This study has dual contributions in theory and practice. Theoretically, this study investigates the spatiotemporal heterogeneity of the HQTD-DE coupling coordination relationship at the urban level. It innovatively employs interpretable machine learning to analyse the non-linear effects of CCD drivers, thereby providing a theoretical framework and research methodology for studies on coupling coordination relationships. In practice, this study is relevant to planners and policymakers, who are concerned about how to deeply integrate the digital economy with high-quality tourism development so as to release new quality productive forces.

## 2. Literature Review

### 2.1. High-Quality Tourism Development

At present, China’s tourism industry continues to maintain a stable growth trend, becoming an important support for the Chinese economic growth, and HQTD has become an important theme under the high-quality development of the Chinese economy. Researchers have conducted numerous studies on the high-quality development of China’s tourism sector, offering valuable insights and references. He and Wang (2020) pointed out that the Chinese tourism industry is undergoing rapid development, but faces low productivity, low end-product supply, and a weak innovation drive, which impede the HQTD [5]. Wei et al. (2016) constructed a systematic evaluation system for HQTD from four aspects: economy, innovation, synergy, and sharing, and found that the high-quality development of tourism is still in the middle-low overall, and the supply of tourism is obviously lagging behind the demand [6]. Ma et al. (2019) evaluated Chinese HQTD from five perspectives: supply, demand, efficiency, operation, and openness, and found that there are regional differences in HQTD, showing a decreasing imbalance from the east to the west [7]. High-quality tourism development is not only determined by economic factors, but also by innovation, efficiency, and synergy etc. Therefore, in many studies, the method of assessing HQTD is mainly based on the construction of a comprehensive evaluation system. In addition, the ecological impacts of tourism should also be included in HQTD, and tourism is currently recognised as one of the contributors to climate warming [8]. The tourism sector affects the global climate at the same time as it is affected by the climate, and the series of environmental problems it causes is becoming increasingly prominent. Li et al. (2022) pointed out that the ecological efficiency of China’s tourism is at a low level, and there is still much room for improvement [9]. However, many HQTD evaluation systems neglect ecologically relevant indicators.

### 2.2. Digital Economy

The concept of the DE was introduced in 1996 [10]. Many scholars have explored its internal logic. The connotation of the DE has shown different characteristics at different historical stages, with early definitions favouring productivity enhancement based on digital technology, as well as emphasising the digital technology industry and its market-oriented application [11,12]. With the deepening of research, the understanding of DE has gradually changed from the economic function of digital technology to the transformation of digital technology on production relations. At present, the DE is defined as economic activities with digital information or data elements as the main resources, with the internet platform as the main information carrier, and with digital technological innovation as the driving force, which are manifested in a variety of new modes and new business forms [13]. Digital information encompasses a variety of carriers, including images, text, and sound, which can be reused [14]. Internet platforms include market-carrying organisations formed by the Internet, such as sharing economy platforms, e-commerce platforms, etc. [15]. Digital technology refers to a new generation of information technology that can process and analyse digital information, such as big data, cloud computing, blockchain, artificial intelligence, etc. [16,17]. At the same time, the DE has given rise to new financial models, such as the new economy of the individual, the unmanned economy, etc. [18]. Therefore, the development of the DE needs to be assessed from multiple dimensions, not only to reflect the degree of support for a region’s DE technology facilities, but also the degree of innovation in digital technology and the development of digital finance.

### 2.3. The Mechanism Regarding the Relationship of Coupling Coordination

In recent years, scholars have conducted rich research on the relationship between tourism development and the digital economy. Related research studies can be categorised into how the digital economy affects tourism development and how tourism development feeds back on the digital economy.

#### 2.3.1. The Effect of DE on HQTD

Tourism development is a powerful tool for economic diversification and social development [19]. Tourism development boosted the local economy, employment, and improved public services [20], and in turn, economic growth and increased income levels have boosted demand for tourism [21]. When people’s income level rises, they will naturally increase their investment in leisure and entertainment, and the digital economy plays an important role in economic and tourism development. Digital technology has overturned the production relations in the tourism industry, and the core of the DE is to use digitised knowledge and information as the key factors of production, and the reproducible, shareable and unlimited growth characteristics of tourism data resources provide a guarantee and a possibility for the integration of the DE, such as online reviews, scenic areas or tourism enterprises can enhance and adjust visitor experiences and marketing strategies [22,23]. Deng et al. (2024) used data from listed tourism companies in China and found that the tourism labour market has undergone significant changes with the deepening digital transformation [24]. Digital transformation has boosted employment growth in companies and increased the demand for highly skilled workers. Xu et al. (2024) illustrated the impact of digital innovation on smart tourism destinations through data mining as well as interviews, providing a new elaboration of the field of innovation in smart tourism destinations [25]. Wu et al. (2023) found that the DE contributes to tourism innovation and the formation of a new pattern of smart tourism, and can accelerate the recovery of the tourism industry and tourism market at a faster pace [26]. Ma and Ouyan (2023) analysed the impact of digital finance on tourism development using panel data from 31 provinces in China, and found that digital finance can improve payment convenience as well as channels to alleviate liquidity constraints, thus significantly contributing to the development of the tourism economy [27]. Li et al. (2024) found that digital finance directly contributes to domestic tourism demand [28]. Therefore, the DE can have an impact on the development of tourism in many ways, such as employment, demand, and innovation.

#### 2.3.2. The Effect of High-Quality Tourism Development on the Digital Economy

While the development of the DE has a huge role in promoting tourism development, the development of tourism is also expected to, in turn, stimulate the advancement of the digital industry. Tourism is a typical representative of the integration and development of the digital economy and the real economy. The application of digital technology not only improves the quality and efficiency of tourism services but also promotes the deep integration and development of the traditional tourism industry and the emerging digital economy industry. Gretzel et al. (2015) pointed out that the tourism industry, as an information-intensive industry, has continued to promote the application and innovation of digital technology [29]. In the tourism industry, digital technology has been widely used in online booking, intelligent guided tours, virtual reality experiences, intelligent travel recommendations, etc., which has promoted the development and application of digital economy technology. Buhalis and Amaranggana (2015) found that providing smart retrofits for tourism destinations promotes the diffusion and application of digital technologies [30]. Xiang and Gretzel (2010) deeply discusses the role of social media in tourism information search and analyses its contribution to the digital economy, and they point out that through social media, tourism enterprises can establish closer ties with potential customers, carry out personalised marketing campaigns, and improve brand exposure and user engagement, while the popularity and wide use of social media provide an important driving force for the development of the DE [31]. Buhalis and Leung (2018) discussed the development of smart hotels and analysed the impact of smart hotels on the DE and its interconnected relationship with other industries [32]. Tourism development provides an important impetus and support for the development of the DE by promoting the application and innovation of digital technology, expanding the market demand of the DE, improving the service quality of the DE, promoting entrepreneurship and innovation in the DE, and promoting the integration of the DE with the real economy. However, it is clear that, in general, the tourism industry has benefited from the development of the DE more than it has contributed to the promotion of the digital economy, because the DE has changed the tourism industry in all aspects, and the tourism industry has expanded the use of digital technology and thus contributed to the development of the digital economy.

In summary, the current research mainly focuses on the unidirectional study of tourism development and DE, but lacks a clear understanding of the interaction between the two systems, and the construction of indicators for high-quality tourism development is still not perfect. In driver analysis, existing studies predominantly rely upon linear assumptions, overlooking the spatio-temporal variations in regional development and the non-linear interactions among relevant factors. This hinders a deeper understanding of the relationship between HQTD and DE. In addition, a large number of studies have focused on Chinese national and provincial levels, with relatively few studies at the city level. Therefore, this study focuses on Chinese prefecture-level cities, constructs a comprehensive evaluation system for high-quality tourism development and digital economy, calculates the CCD of the two systems through CCDM, analyses the coordinated development level of the two systems, and explores the factors driving the coupling coordination degree by using explainable machine learning to supplement the current research gaps.

## 3. Data and Methodology

**Figure 1:**
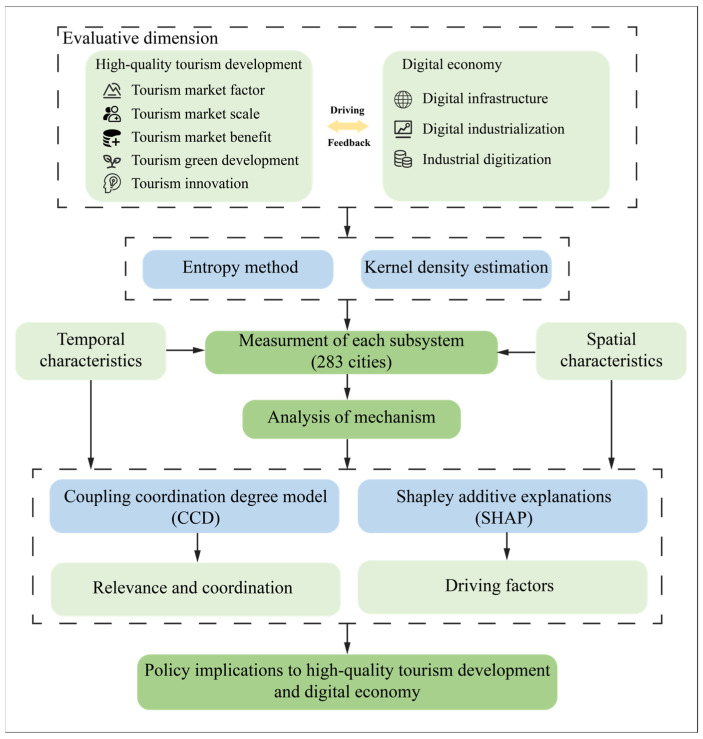
Research framework.

This study conducted an in-depth analysis of the coupling and coordination relationships between the HQTD and DE in Chinese prefecture-level cities, and the main research framework is shown in Figure 1. Based on Figure 1, this section describes each of the research methods.

### 3.1. Study Area

China is a vast country covering an area of 9.6 million square kilometres, encompassing a large number of natural resources and possessing a long history and culture. From north to south and from east to west, each region has its unique characteristics. This provides China with rich tourism resources. With the rapid development of the Chinese economy, the Chinese tourism industry is developing rapidly, and the amount of tourists and tourism revenue in all regions have increased significantly, and with the development of digital technology, its integration with the tourism industry, providing tourists with a more convenient and personalised tourism experience. Due to the unbalanced economic development of Chinese regions, tourism development, especially the HQTD and DE, has shown different degrees of development and matching in different regions, and exploring such temporal and regional differences can help to provide a better understanding, which can guide the development of tourism resources in each region according to local conditions.

**Figure 2:**
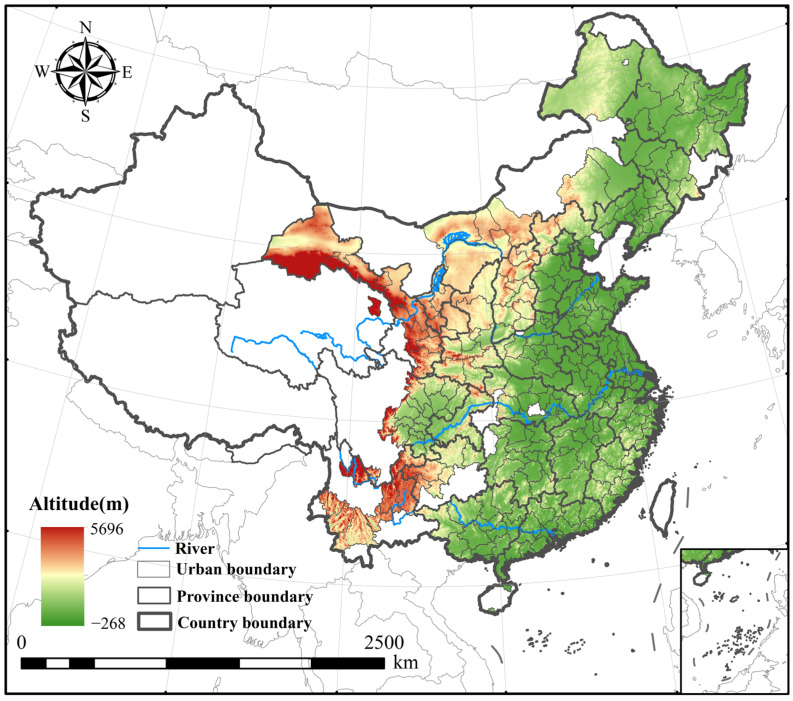
Study area of this study.

Therefore, this study selects 283 cities as the research sample to fully explore this spatiotemporal variation, as shown in Figure 2. These samples include 279 prefecture-level cities across China, as well as the four municipalities directly under the central government (Beijing, Shanghai, Tianjin, and Chongqing). Some regions, such as autonomous states, are excluded due to insufficient data. When data are available, the study scale is higher than that of provincial administrative units, allowing for a better representation of regional differences.

### 3.2. Index System and Data Sources

HQTD represents the unity of quantity and quality, speed and efficiency, requiring continuous enhancement of quality on the basis of quantity. It is necessary not only to pursue sustainable and balanced growth, but also to focus on conducting comprehensive assessments and value evaluations of tourism development quality, encompassing economic efficiency, industrial structure, and environmental quality. Based on the comprehensive survey in the literature review, this study conducts a comprehensive assessment of HQTD across five dimensions: tourism market factor, tourism market scale, tourism market benefits, tourism green development, and tourism innovation.

#### 3.2.1. Tourism Market Factor

Tourism market factor reflects the overall demand-side environment that shapes tourism development, emphasising the conditions and characteristics of the tourism market that influence the growth and quality of tourism activities [33]. It focuses on how visitor demand, spending behaviour, and market composition collectively determine the potential for destinations to provide high-quality tourism experiences [34]. By highlighting the market’s structure and dynamics, this dimension illustrates how favourable demand conditions contribute to sustainable development, service improvement, and enhanced competitiveness, providing the theoretical foundation for understanding high-quality tourism growth.

#### 3.2.2. Tourism Market Scale

Tourism market scale captures the size and structural composition of the tourism market, which directly reflects the potential demand that sustains tourism growth [35,36]. It emphasises both the overall volume of tourism activities and the diversity of visitor segments, highlighting how a large and well-structured market supports HQTD [37,38]. For instance, the total number of tourists indicates the market’s capacity, while the distribution between domestic and international visitors reflects market diversity. Together, these aspects illustrate how the scale and structure of the market underpin development potential and the ability of destinations to respond to evolving visitor preferences.

#### 3.2.3. Tourism Market Benefit

Tourism market benefit emphasises the economic value generated by tourism activities and its role in supporting high-quality development [39,40]. It reflects how tourism contributes to local economies, promotes employment, and enables investment in infrastructure and service improvements [41]. The economic performance of the tourism market demonstrates its capacity to sustain growth and enhance the overall quality of tourism experiences. Observations of revenue patterns and spending behaviour can help illustrate how effectively tourism translates demand into tangible economic outcomes, highlighting the link between financial performance and the advancement of HQTD.

#### 3.2.4. Tourism Green Development

Tourism green development reflects the extent to which tourism growth aligns with environmental sustainability and ecological protection [42,43], which is a key dimension of high-quality tourism. It emphasises minimising negative environmental impacts while promoting efficient use of natural resources, ensuring that tourism activities contribute positively to the overall ecological environment [28]. Green development in tourism not only supports the long-term viability of destinations but also enhances the quality of visitor experiences by maintaining clean, safe, and aesthetically pleasing environments [44,45]. Environmental performance, such as effective waste management, pollution control, and preservation of green spaces, can serve to illustrate how destinations integrate sustainability into tourism development, reinforcing the role of ecological considerations in achieving high-quality tourism outcomes.

#### 3.2.5. Tourism Innovation

Tourism innovation reflects the capacity of the industry to generate new knowledge, technologies, and creative solutions that enhance the quality, diversity, and competitiveness of tourism [25,46]. It emphasises how innovative practices can improve visitor experiences, optimise service delivery, and support the long-term sustainability of tourism destinations [47,48]. Innovation drives the transformation of tourism products, encourages the integration of new technologies, and fosters continuous improvement, all of which are essential for achieving HQTD. This dimension highlights the central role of innovation in shaping dynamic, competitive, and sustainable tourism.

Considering the data availability at the city level, specific secondary indicators are constructed as shown in Table 1. Data for each indicator can be obtained from the China Statistical Yearbook, the China Urban Statistical Yearbook, the Statistical Bulletin on National Economic and Social Development of the People’s Republic of China, and the Statistical Bulletin on National Economic and Social Development of various cities. For the calculation of tourism carbon emissions in each city, an “up-bottom” method is adopted [49], carbon emission accounts and datasets (CEADs) [50] are firstly used to collect the carbon emissions from the tertiary industry of each prefecture-level city, and the tourism carbon emissions are converted by the ratio of the added value of the tourism industry in the tertiary industry to obtain the carbon emissions from the tourism industry in each city.

According to Liu et al. (2020) [51] and He and Pan (2021) [52], five indicators are chosen to measure the DE, including the digital infrastructure, digital industrialisation, industrial digitisation, and digital technology support of each city, and the data are obtained from the statistical bulletin of the national economic and social development of each city, as well as the Peking University digital inclusive finance index [53]. For the few missing values, linear interpolation is employed [54,55,56], whereby the missing data are filled by linear trends.

**Table 1:** Evaluation index system of the relationship between HQTD and DE.

Subsystem	Dimension	Indicators	Indicator Type
High-quality tourism development	Tourism market factor	Number of scenic spots of A level and above [57]	+
		Total travel agencies (number) [58]	+
		Total star-rated hotels (number) [58]	+
		Number of tourism employees (10,000 person-times) [59]	+
	Tourism market scale	Total number of tourists (10,000 person-times) [42]	+
		Number of domestic tourists (10,000 person-times) [42]	+
		Number of international tourists (10,000 person-times) [60]	+
	Tourism market benefit	Earnings from domestic tourism (100 million yuan) [61]	+
		Foreign exchange earnings from international tourism (USD 10,000) [61]	+
		Total tourism earnings (100 million yuan) [62]	+
		Per capita tourist spending (yuan) [62]	+
		Proportion of total earnings from tourism in GDP (%) [41]	+
		Percent of total earnings from tourism in tertiary industry (%) [38]	+
	Tourism green development	Tourism carbon emission(million ton) [48]	−
		Sewage treatment rate (%) [63]	+
		Green coverage in urban built-up areas (%) [63]	+
		Harmless treatment rate of household garbage (%) [63]	+
	Tourism innovation	Tourism industry research and development fund investment (million yuan) [63]	+
		Number of tourism patents granted (number) [43]	+
		Number of tourism R&D personnel [64]	+
Digital economy	Digital infrastructure	Internet access port density (number/person) [51]	+
		Cell phone base station density (number/km^2^) [51]	+
	Digital industrialisation	The proportion of information-based employees (%) [52]	+
		Total telecommunications services per capita (yuan/person) [52]	+
	Industrial digitization	Digital Financial Inclusion Index [52]	+

+ is the positive indicator, − is the negative indicator

### 3.3. Entropy Method

Before calculating the weights for each indicator, the data in the indicators need to be normalised due to the different scales. In this study, maximum-minimum normalisation is used, with positive indicator Equation (1) and negative indicator Equation (2). *x_i*,*j_* is the *j*th indicator value for the *i*th city. *min*(*x_i*,*j_*) is the minimum value of the *j*th indicator. *max*(*x_i*,*j_*) is the maximum value of the *j*th indicator

(1) $$T_{i,j}=\frac{x_{i,j}-min\left(x_{i,j}\right)}{max\left(x_{i,j}\right)-min\left(x_{i,j}\right)}$$

(2) $$T_{i,j}=\frac{max\left(x_{i,j}\right)-x_{i,j}}{max\left(x_{i,j}\right)-min\left(x_{i,j}\right)}$$

Determining the weights of the indicators in the constructed indicator system is an important part of the coupled coordination analysis. In this study, the entropy weighting method was used to determine the weights of each indicator. Entropy weighting is an objectivity weighting method which is completely data-driven and easy to compute compared to the subjective weighting method, where the weights are determined by human beings, and has been used in many studies [65,66]. The entropy weighting method calculates the information entropy of each indicator and converts the information entropy into weights to reflect the importance of each indicator to the result [67]. The steps for calculating the entropy weight method are as follows:

(3) $$P_{i,j}=\frac{T_{i,j}}{\sum_{i=1}^{m}T_{i,j}}$$

where *P_i*,*j_* denotes the proportion of indicator j to the total.

(4) $$e_{j}=-\frac{1}{ln\;m}\sum_{i=1}^{m}\left(P_{i,j}\times lnP_{i,j}\right)$$

where *e_j_* represents the information entropy of indicator *j*. A larger *e_j_* indicates a smaller difference coefficient of the indicator.

(5) $$g_{j}=1-e_{j}$$

where *g_j_* represents the difference coefficient of indicator *j*, with a larger difference coefficient indicating that the indicator is more important.

(6) $$w_{i}=\frac{g_{j}}{\sum_{j=1}^{n}g_{j}}$$

The weight of each indicator is calculated by taking the difference coefficient of each indicator as a proportion of the total difference coefficient. In order to reflect the variability of the evaluation results in different years, the weights were calculated by pulling through the indicator data for all years.

### 3.4. Coupling Coordination Degree Model

In this study, the CCDM is applied to explore the interactions between HQTD and DE, which reflect the degree of synchronised development between the two systems. The higher the value of the coupling coordination degree, the higher the synergy, indicating a considerable level of development. The formulas for coupling coordination degree are as follows:

(7) $$D=\sqrt{C\times T}$$

(8) $$C={\left(\frac{HQTD\times DE}{{\left(\left(HQTD+DE\right)/2\right)}^{2}}\right)}^{\frac{1}{2}}$$

(9) T=αHQTD+βDE

where *D*($D\in \left[0,1\right]$) represents the coupling coordination degree, *C* represents the coupling degree of high-quality tourism development and digital economy, and *D* represents the comprehensive evaluation index. α and β denote the weights between combinations of indicators, generally α = β *=* 0.5 [68]. Since there are no uniform standards for classifying CCD, CCD is categorised into five types based on similar research [66,69], as shown in Table 2.

### 3.5. Kernel Density Estimation

For exploring the characteristics of the evolution in temporal coordination of the coupling between high-quality tourism development and the digital economy over time. Kernel density estimation is a nonparametric statistical method for estimating the unknown form of the probability density function, which does not require the assumption that the unknown variable needs to conform to a defined distribution. It estimates the probability density function by placing a kernel function (e.g., a Gaussian kernel function) around each data point and estimating the probability density function by taking a weighted average of them, thus estimating the probability density function of a random variable more accurately. The formula is as follows [70]:

(10) $$f\left(x\right)=\frac{1}{m}\sum_{i=1}^{m}K_{h}\left(x-x_{i}\right)$$

In the above equation, *m* is the number of cities, totalling 283, *h* is the bandwidth of the density estimation, and *K* is the chosen kernel function. The larger the bandwidth, the smoother the density function will be; the most commonly used Gaussian kernel function is used.

### 3.6. Shapley Additive Explanation (SHAP)

Although certain tree-based machine learning models, such as Random Forests, XgBoost, and LightGBM, offer a viable method for uncovering the non-linear relationships between features and outputs. However, these models only provide aggregate results and cannot capture the non-linear effects of individual features [71]. Therefore, this study employs SHAP to enhance the interpretability of machine learning model results. The SHAP method draws upon the Shapley value from game theory [72] to assess the relative importance of individual features within the model. SHAP calculates the Shapley value for each feature by considering all possible combinations, thereby revealing the marginal contribution of each variable to the model’s output [73]. This method is applicable not only to global interpretation but also to local interpretation. Making it an important tool for model interpretability. The equations are as follows:

(11) $$\varphi_{i}=\sum_{S\subseteq H\backslash \left\{i\right\}}\frac{\left|S\right|!\left(\left|H\right|-\left|S\right|-1\right)!}{\left|H\right|!}\left(t\left(S\cup \left\{i\right\}\right)-t\left(S\right)\right)$$

where $\varphi_{i}$ denotes the SHAP value for driving factor *i*, *H* represents the overall feature set, *S* denotes a subset of *H* excluding feature *i*, *t*(*S*) denotes the model prediction result for the feature combination within subset *S*. Furthermore, nine machine learning algorithms are selected, including decision trees, random forests, k-nearest neighbour regression, support vector machines, LightGBM, CatBoost, XgBoost, AdaBoost, and GBDT. Optimal hyperparameters for each model are determined via grid search using 5-fold cross-validation. The model demonstrating the highest performance is ultimately chosen for SHAP interpretability learning. All models are implemented using Python 3.10.16.

## 4. Results and Discussion

### 4.1. Evaluation of High-Quality Tourism Development and Digital Economy

**Figure 3:**
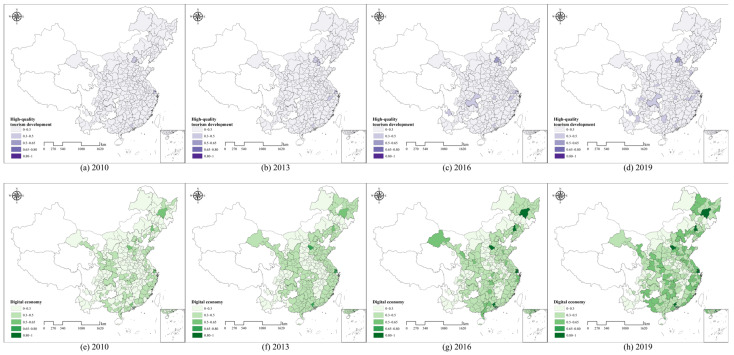
The comprehensive evaluation results of high-quality tourism development and the digital economy. (**a**–**d**) High-quality tourism development, (**e**–**h**) digital economy.

**Figure 4:**
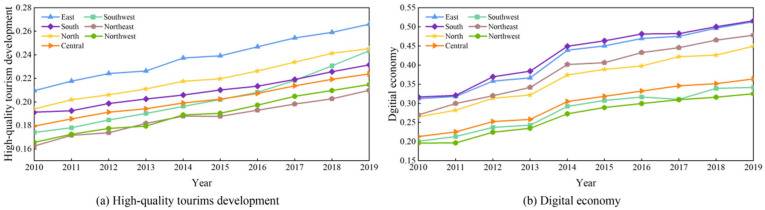
Level of high-quality tourism development and digital economy from 2000 to 2019.

The results of the comprehensive evaluation of HQTD and DE in Chinese prefecture-level cities are shown in Figure 3a–d, which display the results for the years 2010, 2013, 2016, and 2019. Figure 4a illustrates the trends of HQTD and DE across different regions. According to Figure 3a–d, cites with higher levels of HQTD are mainly concentrated in developed regions. In 2010, only three cities, Beijing, Shanghai, and Guangzhou, exceeded 0.3. By 2019, HQTD levels in these cities continue to rise, with Beijing exceeding 0.6. Other cities with notable increases in HQTD are mainly provincial capitals or municipalities directly under the Central Government, such as Hangzhou, Chengdu, Kunming, Wuhan, Chongqing, and Tianjin, as well as tourist-rich cities like Lijiang and Huangshan. Overall, the level of HQTD remains relatively low across most cities in China. This is largely because tourism development during 2010–2019 is primarily measured in terms of economic benefits, with less focus on the broader development of high-quality tourism. However, Figure 4 indicates HQTDs across all regions are increasing annually. The HQTD is highest in the western region, followed by the northern and southern regions. Before 2016, HQTD levels in the southwestern region remained at a medium-to-low level but rose rapidly thereafter. This spatial imbalance is consistent with existing research showing that eastern and coastal regions, supported by stronger economies and industrial structures, generally achieve higher levels of tourism development [74]. Compared with earlier studies, the present analysis highlights intra-regional heterogeneity and shows that western cities have accelerated since 2016, reflecting the role of emerging industries and policy support [75]. These findings suggest that regional disparities remain pronounced, yet the dynamic growth in the west indicates a gradual narrowing of the gap. Compared with studies focusing on provincial or national scales [76], the city-level analysis in this study provides more granular insights, revealing intra-regional heterogeneity in digital economy development.

Figure 3e–h demonstrates that from 2010 to 2019, the level of DE development in most Chinese cities markedly improved. The Yangtze River Delta, Pearl River Delta, and Bohai Bay regions exhibit high levels of DE. In 2019, the DE exceeded 0.5 in all cities within the Yangtze River Delta region, with Shanghai reaching 0.9. These cities with high DE development level are more economically developed and have better industrial support, forming urban clusters with regional centre cities as the core, which provides a favourable environment for the development of the digital economy. This spatial pattern aligns with prior studies highlighting that coastal cities benefit from historical economic advantages, strong industrial bases, and urban agglomeration effects [77,78]. These findings indicate that economic prosperity and industrial support remain critical drivers of regional digital transformation, reinforcing the role of urban centrality in shaping digital development outcomes. The DE of cities within the Chengdu–Chongqing economic circle in western China experienced a rapid increase from 2010 to 2019. The central provinces, including Jiangxi, Anhui, and Henan, exhibit relatively underdeveloped DE indicators, with values predominantly below 0.3. This may be attributable to the absence of fully established digital industries in these regions, coupled with the lack of a particularly dominant central city. Figure 4b demonstrates that DE exhibits varying degrees of growth across different regions. A comparison between HQTD and DE reveals that high-quality tourism development lags significantly behind the digital economy. Subsequent sections provide a detailed analysis of the degree of coupling and coordination between HQTD and DE.

### 4.2. Coupling and the Coordinated Relationship Between HQTD and DE

**Figure 5:**
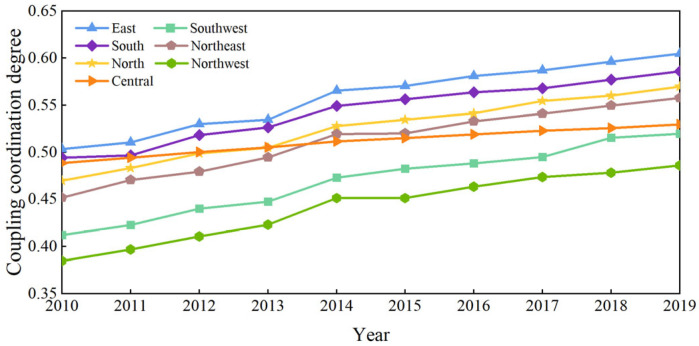
Level of CCD from 2000 to 2019.

Figure 5 demonstrates the average coupling coordination degree (CCD) between the HQTD and DE across different regions from 2010 to 2019. While the CCD shows an overall upward trend during this period, regional variations are evident. The CCD in the eastern region is consistently above 0.5, maintaining a bare balanced status, with a pronounced upward trend, reaching an average CCD of nearly 0.6 in 2019. The trend in CCD values in the southern region is similar to the eastern region, though the values are slightly lower. The CCD growth in the central region is the lowest, remaining at approximately 0.5 throughout the entire study period. The CCD in the northwest region is the lowest, at only 0.45 in 2019, primarily due to the underdevelopment of both the tourism and digital industries in the region.

**Figure 6:**
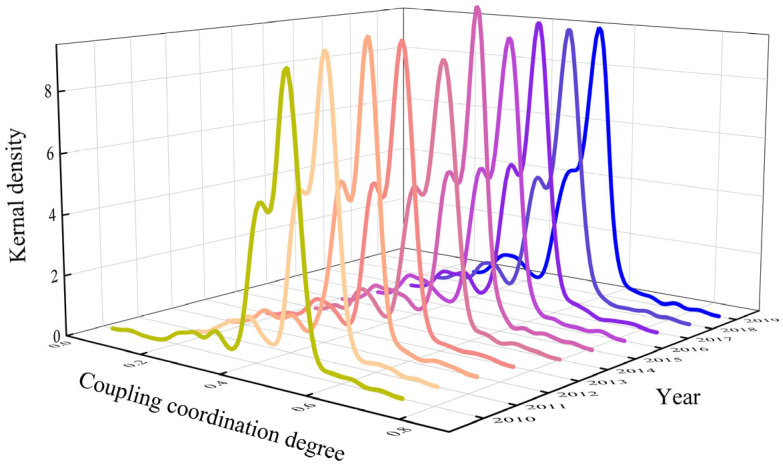
Kernel density estimation curve of CCD.

The distribution of the CCD from 2010 to 2019 is fitted by the kernel density estimation, as shown in Figure 6. Figure 6 demonstrates the distribution location, pattern, distribution, and polarisation trend of the CCD. From 2010 to 2019, the centre of the kernel density curve is more to the left and shows a trend of leftward bias, which fully illustrates that the DE has not yet effectively promoted the HQTD, consistent with studies showing that the impact of digital economy on tourism is still uneven across regions and sectors, and that the integration of digital technologies into tourism services and management remains limited [79]. Additionally, the kernel density curve displays a distinct trailing effect, which results from the presence of a small number of cities with either very low or very high CCD levels. This reflects a significant gap in the coordinated development of HQTD and DE at the city level. The sharp distribution pattern of the curve indicates that the discrete development status between HQDT and DE has not been improved.

#### 4.2.1. Spatial Analysis of Coupling Coordination Degree

**Figure 7:**
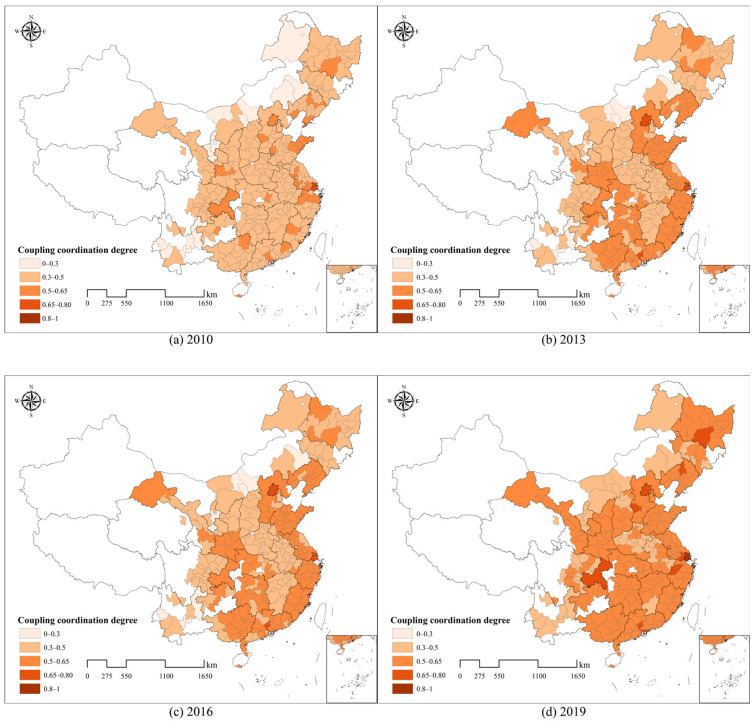
The 2010, 2013, 2016, and 2019 spatial and temporal evolution of CCD.

The spatial and temporal evolution of the CCD is shown in Figure 7. As illustrated, the CCD in China exhibits an overall upward trend over the study period, though significant regional heterogeneity exists. In 2010, high CCD values were primarily concentrated in the core urban areas of the Yangtze River Delta, centred around Shanghai, and the Shandong Peninsula, with sporadic high values in other cities across the south, north, and southwest. These include provincial capitals such as Guangzhou, Xi’an, Wuhan, Chongqing, and Beijing. However, the overall level of CCD was relatively low in 2010, with most cities falling within the 0.3–0.5 range, reflecting a slightly unbalanced status. By 2013, with the exception of the central provinces of Jiangxi, Anhui, Henan, and Shanxi, as well as the southwestern provinces of Sichuan and Yunnan, the CCD of cities in other regions improves, with many surpassing 0.5 and achieving a barely balanced status. Notably, Shanghai and Beijing exceed 0.65. By 2016, more cities reached a barely balanced status, and several provinces saw their CCD surpass 0.5, including Guangdong. In 2016, cities in Guangdong, Fujian, Zhejiang, Jiangsu, Shandong, Liaoning, and Gansu all exceeded 0.5. This trend reflects the faster development of tourism and the digital economy in these coastal provinces compared to inland areas. In particular, Guangzhou, Shenzhen, Shijiazhuang, Shenyang, and Harbin all exceed 0.65, with each being a provincial capital. By 2019, only Shanghai achieves a superior balanced status, while most other cities remain barely balanced.

The spatial evolution of the CCD in Figure 7 shows that cities with higher CCD values within a given region typically emerge first in provincial capitals or regional centres, then radiate to surrounding cities. For instance, in the Yangtze River Delta, cities in Jiangsu, Zhejiang, and Shanghai initially exhibit high CCD, which gradually spreads across the entire Jiangsu and Zhejiang provinces from 2010 to 2019, forming clusters of high-value CCD cities. This pattern is also evident in the Pearl River Delta, the Shandong Peninsula, and the Bohai Rim. This reflects a high degree of spatial autocorrelation. To test this, the global Moran index [70] is applied to analyse the aggregation characteristics of CCD. The global Moran index measures the overall spatial distribution of correlation by comprehensively considering both the variation in variable values and spatial proximity relationships. Table 3 presents the statistics for the global Moran index across the years, confirming a significant positive spatial correlation for the CCD from 2010 to 2019, which supports the idea that coupling coordination spreads from central cities to surrounding areas.

#### 4.2.2. Analysis of Coupling Coordination Degree Types

**Figure 8:**
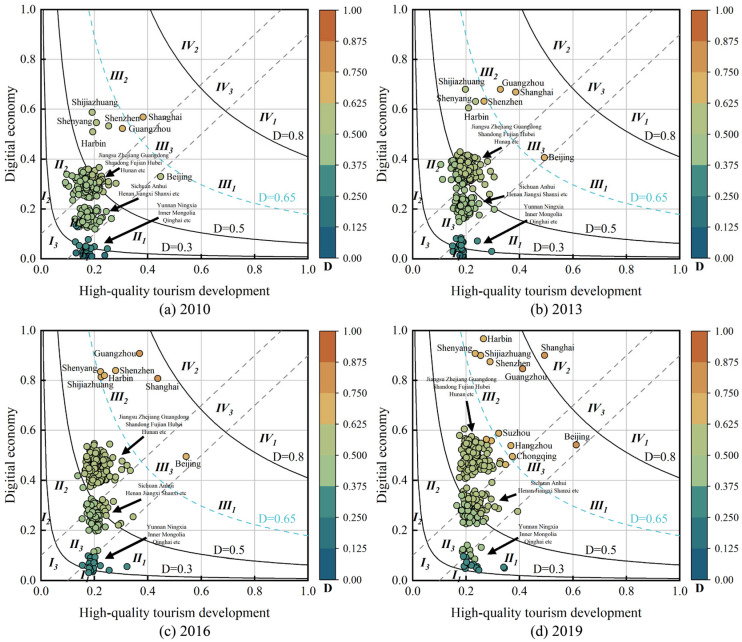
The 2010, 2013, 2016, and 2019 CCD types. D represents coupling coordination degree.

According to the CCD classification method proposed in Section 3.4, the coupling coordination results from 2010 to 2019 are categorised as shown in Figure 8. Most of the cities in 2010 are in extreme unbalanced, while some of the cities in extreme unbalanced are mainly concentrated in Yunnan, Ningxia, and Inner Mongolia. In cities with extreme unbalanced status, the development of DE lags significantly behind HQDT. Other cities in the slightly unbalanced status lag behind in the development of HQTD compared to DE. In 2010, only the city of Shanghai achieved a bare balance. The scatter points in Figure 8 exhibit distinct clustering, primarily attributable to variations in DE. In 2013, the status of CCD improved. Compared with 2010, the CCD in most cities is primarily driven by the improvement in DE, while the level of improvement in HQTD remains low. The clustering in CCD is more pronounced in 2016 and 2019. The lag in HQTD is further exacerbated, leading to many cities remaining in an unbalanced status. Cities in bare balanced status primarily belong to the III2 type, where their DE exceeds 0.8 but HQDT remains below 0.4. Only Beijing maintains a balanced development pattern across both systems. This further demonstrates that HQTD and DE exhibit a relatively low degree of correlation in most cities, with the lagging development of HQTD hindering the synergistic advancement of the systems.

### 4.3. Relative Importance of CCD Drivers

**Figure 9:**
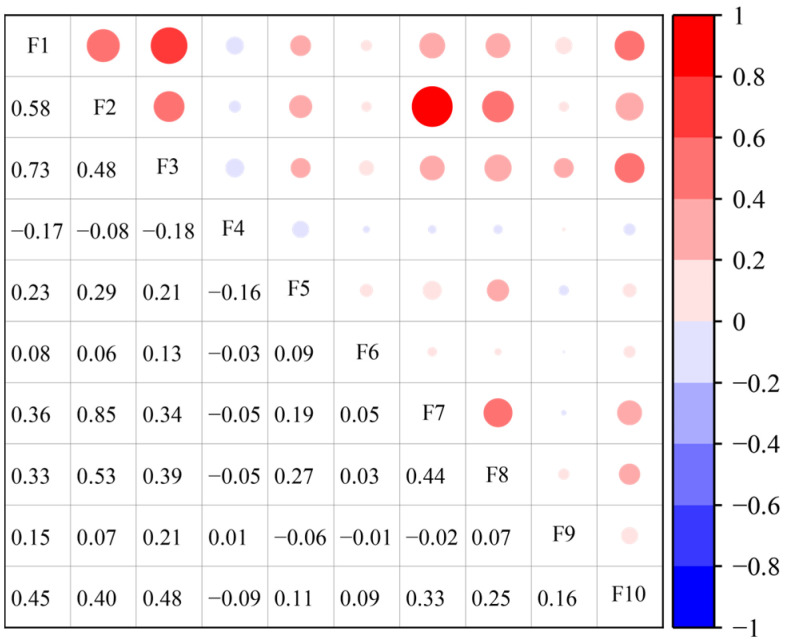
Matrix of correlation coefficients of driving factors.

SHAP is used in this study to analyse the drivers influencing the CCD. Based on the availability of data at the city level, as well as avoiding and selecting the duplication of indicators in the previous evaluation system, 10 indicators in Table 4 are selected for analysis from three aspects: economic, social, and labour force. These factors are highly relevant to the development of high-quality tourism and the digital economy [27,62,80], and can promote their synergistic development to varying degrees, and the correlation coefficients of these indicators are shown in Figure 9. Only the per capita GDP and total social retail sales per capita have a strong correlation with other factors, and this strong covariance will cause a great deal of the results in the usual linear regression. Drivers as the input variable X, and coupling coordination as the output variable Y, with data split into training and test sets in the 8:2 ratio. The optimal parameters for nine machine learning models are determined through 5-fold cross-validation and grid search. The results for each model are presented in Table 5. Based on the results, LightGBM achieved the highest R^2^ value of 0.7294, demonstrating the best predictive performance. Consequently, LightGBM is selected for SHAP explainability analysis.

**Figure 10:**
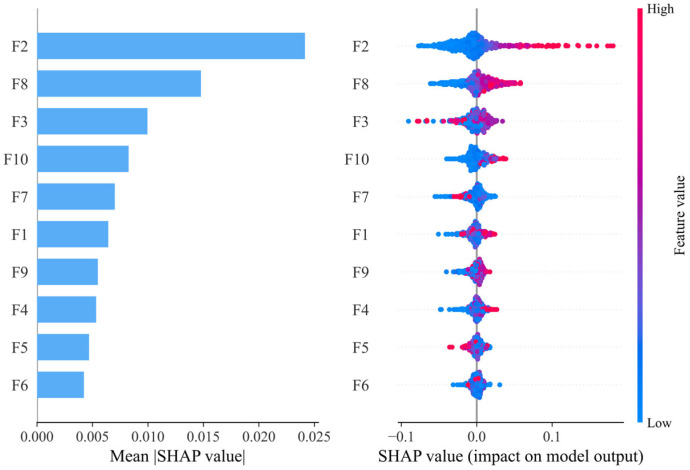
The importance of CCD drivers.

Figure 10 presents the ranking of SHAP values for the CCD drivers. F2 is identified as the most important driver, followed by F8. Highlighting the pivotal role of consumption and the tertiary sector in promoting the synergistic development between high-quality tourism development and the digital economy. The swarm plot in Figure 10 further indicates that higher levels of social retail sales and proportion of the tertiary sector correlate with higher CCD values, and lower values are associated with lower CCD. This finding echoes the literature emphasising that tourism and digital economy coordination is strongly shaped by dynamics on the demand side [63]. High consumption levels generally indicate greater spending power among tourists, thereby boosting tourism development. In the context of the digital economy, digital tools such as e-commerce, online payments, and smart retail enhance consumer convenience and efficiency, making it easier to expand the sales of tourism-related products and services. The high proportion of the tertiary sector indicates a mature service industry, and the optimised industrial structure provides a solid economic and service foundation for tourism. The impact of F3 on CCD exhibits a certain degree of non-linearity, with higher F3 leading to lower CCD. This phenomenon is likely to occur in resource-based cities, where increases in urbanisation rates drive industrial development rather than the tertiary sector. The impact of digital R&D intensity on CCD is minimal, likely due to the lag effect inherent in R&D activities.

**Figure 11:**
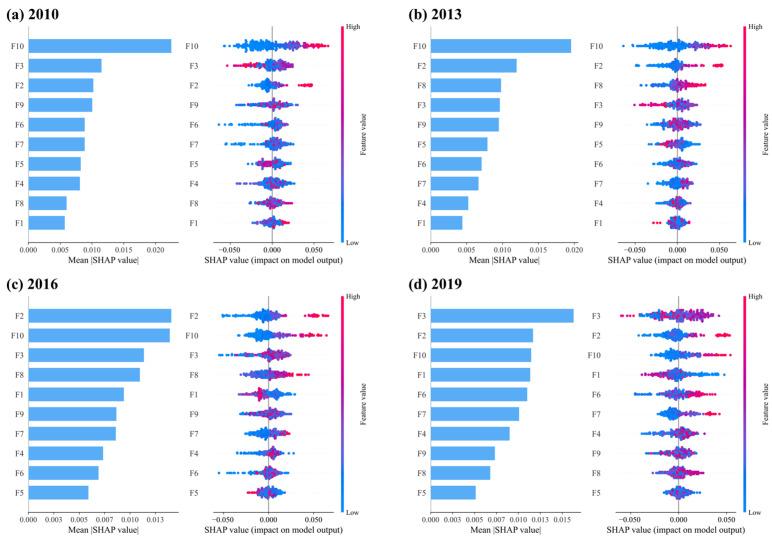
The importance of CCD drivers in different years.

Furthermore, the spatio-temporal heterogeneity of CCD drivers is analysed. Figure 11 presents the SHAP results for the drivers in 2010, 2013, 2016, and 2019. The results demonstrate a consistent trend. With the exception of 2013, the importance of F10, F2, and F3 consistently ranks among the top three. In 2010 and 2013, F10 exhibits the highest importance with an average SHAP value approaching 0.2, while the second-ranked variable is only around 0.1. In 2010 and 2013, F10 had the highest importance, with an average SHAP value approaching 0.2, while the second-ranked feature only achieved around 0.1. From 2010 to 2015, China accelerated its integration into global markets. The expansion of trade not only facilitates the movement of goods but also fosters international exchanges and personnel flows, thereby creating favourable conditions for inbound tourism. In 2016 and 2019, F10’s significance declined and was surpassed by F2 and F3. Following 2015, China’s economy entered a new normal, placing greater emphasis on domestic demand-driven growth. National strategy gradually shifts from “export-led growth” towards “consumption upgrading” and “new urbanisation”. Consequently, the explanatory power of urbanisation rates and social retail sales surpasses the percentage of total exports and imports.

F3 exhibits a non-linear relationship with CCD across all years, highlighting the differences in development patterns between various cities. F1’s importance increases annually, from last to fourth in 2019. However, F1 and CCD primarily exhibit a negative correlation, with only a few cities showing a positive relationship. Suggesting a threshold effect or U-shaped relationship may exist between economic development and CCD. This regional heterogeneity indicates that consumption demand, industrial structure, and basic economic capacity play differentiated roles in shaping the coordination between digital economy and high-quality tourism development. These findings are consistent with previous studies emphasising that the effectiveness of digital empowerment in tourism is strongly conditioned by regional development stages and resource endowments [18,81]. Therefore, region-specific strategies are required to enhance the synergy between the digital economy and high-quality tourism development.

**Figure 12:**
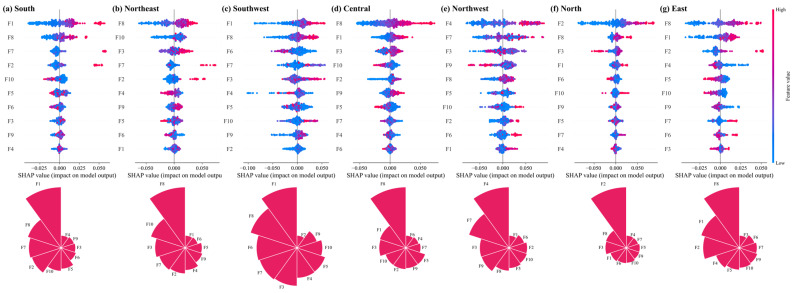
The importance of CCD drivers in different regions.

Figure 12 illustrates the results of the importance assessment of regional heterogeneity. These drivers vary significantly across regions, reflecting the unique characteristics and challenges of each region in terms of coupling and coordinating high-quality tourism development and the digital economy. F2 exhibits greater importance in the northern and eastern regions, with values of 0.0287 and 0.0101, respectively. These two regions are more economically developed, dominated by urban tourism, and therefore more affected by demand pull. The growth of total social retail sales reflects the purchasing power and consumption demand of consumers; thus, its importance is highlighted in these regions. F8 holds greater importance in all regions except the northwest, indicating that the development of the tertiary sector plays a significant role in CCD across most cities. F4 and F3 hold a greater importance in the northwest, both being highly correlated with labour capital. The region’s low population density and lack of talent in tourism and digital technology hinder its coordinated development. F1 exhibits a negative impact at the aggregate sample level yet demonstrates a predominantly positive promotional effect at the regional level. This discrepancy may be attributed to reduced sample size, coupled with the inherent convergence of economic development within a given region, which prevents the detection of non-linear relationships at the regional level.

## 5. Conclusions and Policy Implications

This study first constructs a comprehensive evaluation index for HQTD and DE based on the available data of tourism development and digital economy in Chinese prefecture-level cities from 2010 to 2019. Subsequently, the CCDM is used to analyse the spatio-temporal evolution trend of the CCD of HQTD and DE. Further, explainable machine learning is employed to analyse the drivers of CCD. The main conclusions of the study are as follows:

(1) The development levels of HQDE and DE in Chinese cities show a gradual upward trend from 2010 to 2019, rising nationally from 0.1807 and 0.2434, respectively, in 2010 to 0.2318 and 0.4113 in 2019. Among them, Beijing, Shanghai, and Guangzhou have a high level of HQTD, while almost all other cities are below 0.5. DE exhibits a higher level of development in the eastern and southern regions. The central, northwestern, and southwestern regions demonstrate a markedly lower level of DE development. Furthermore, a significant disparity exists between the development levels of HQTD and DE.
(2) The level of CCD rises annually, yet exhibits significant temporal and spatial heterogeneity. The cities with the lowest CCD values are mainly concentrated in the northwest region, where the imbalance has persisted. All cities in the eastern region have a CCD above 0.5, indicating a balanced status. The core city of Shanghai exceeded 0.8 in 2019. CCD exhibits a characteristic of spreading outwards from central cities, reflecting the clustering character of tourism and digital economic development. HQTD significantly lags behind the development of DE, which hinders the transition of many cities’ CCDs from incoordination to coordination.
(3) Total social retail sales per capita and percentage of the tertiary sector exert a strong positive driving impact on CCD. Consumption holds greater importance for cities in the north and east. The development of the tertiary sector has a positive impact on CCD in most cities. Labour capital has a higher importance in cities within the northwest region. It is also found that the level of economic development and the urbanisation rate exhibit a non-linear impact on CCD. These findings underscore the need for tailored emission reduction strategies that account for the spatial and temporal diversity of urban tourism and the digital economy.

Based on the above findings, this study makes the following recommendations for the development of high-quality tourism and the digital economy in China:

(1) Chinese tourism resources should accelerate the formulation of policies related to high-quality development of tourism to provide a stronger driving force for high-quality development of tourism, and need to gradually spread from large cities to small and medium-sized cities, especially ecotourism and sustainable tourism, thereby reducing tourism carbon emissions and minimising the ecological impact. Further accelerate the development of the digital industry, not only the tourism industry, but also digital technology is crucial to the development of the whole industry, especially in the central region, where the development of the digital economy lags behind, and needs to be strengthened in the construction of the digital industry.
(2) The application of digital services, technology, and finance in the tourism industry needs to be accelerated. At present, the overall level of coupling coordination in China is not high and lags behind the pace of high-quality tourism development. Therefore, cities should simultaneously promote the development of tourism and the integration of the digital industry. Digital services can enhance the customer’s tourism experience; digital technology provides more diversified travel options and enables companies to develop new tourism products; and digital finance offers both firms and customers multiple channels of financing and payment. There is also a need to further strengthen the spreading influence of provincial capitals and regional centres in the surrounding areas.
(3) According to regional heterogeneity, different measures should be adopted for different regions. Economically developed regions such as eastern, northern, and southern China should continue to develop their tertiary industries, which can effectively promote the development of tourism and the digital economy. For economically backward regions such as Southwest and Northwest China, priority should be given to developing the local economy and increasing the local labour force.

This study contributes to academic research in several ways. By framing the interaction between the digital economy and high-quality tourism through a coupling coordination perspective, it extends theoretical understanding of how these two domains influence each other. The combination of a comprehensive evaluation system with machine learning analysis provides a novel methodological tool that can be applied to other regional and sectoral studies, enhancing the rigour and versatility of empirical investigations. Moreover, the findings on spatial patterns and driving factors offer empirical evidence that validates theoretical assumptions and informs future research and policymaking, thereby establishing a foundation for advancing both theory and practice in the field. However, there are still some issues that deserve further research, and the evaluation system we constructed may be lacking due to the accessibility of city-level data, which is worth remedying in future research. Furthermore, while explainable machine learning offers certain insights and explanatory power regarding the drivers of CCD, the causal relationships within it require further validation.

## Figures and Tables

**Table 2:** Classification of the coupling coordination degree.

D	Categories	Development Balance	Subcategories	Abbreviation
0 < D < 0.3	Extreme unbalanced	HQTD − DE > 0.1	Extreme uncoordinated with lagging high-quality tourism development	I1
		DE − HQTD > 0.1	Extreme uncoordinated with digital economy	I2
		|HQTD − DE| ≤ 0.1	Extreme uncoordinated between high-quality tourism development and digital economy	I3
0.3 < D ≤ 0.5	Slight unbalanced	HQTD − DE > 0.1	Slight uncoordinated with lagging high-quality tourism development	II1
		DE − HQTD > 0.1	Slight uncoordinated with digital economy	II2
		|HQTD − DE| ≤ 0.1	Slight uncoordinated between high-quality tourism development and digital economy	II3
0.5 < D ≤ 0.8	Bare balanced	HQTD − DE > 0.1	Bare coordinated with lagging high-quality tourism development	III1
		DE − HQTD > 0.1	Bare coordinated with digital economy	III2
		|HQTD − DE| ≤ 0.1	Bare coordinated between high-quality tourism development and digital economy	III3
0.8 < D ≤ 1	Superior balanced	HQTD − DE > 0.1	Superior coordinated with lagging high-quality tourism development	IV1
		DE − HQTD > 0.1	Superior coordinated with digital economy	IV2
		|HQTD − DE| ≤ 0.1	Superior coordination between high-quality tourism development and digital economy	IV3

**Table 3:** Global Moran’s I index of coupling coordination degree from 2010–2019.

	Year	2010	2013	2016	2019
Coupling coordination degree	P	0.0000	0.0000	0.0000	0.0000
	Z	12.6007	14.2660	13.5850	13.6094
	Moran’I	0.2556	0.2905	0.2765	0.2772

**Table 4:** Driving factor selection of coupling coordination degree of high-quality tourism development and digital economy.

Driving Factor	Unit	Abbreviation	Source
Per capita GDP	CNY	F1	China Statistical Yearbook, Statistical Bulletin of the People’s Republic of China on National Economic and Social Development, Statistical bulletin on the national economic and social development of cities
Total social retail sales per capita	CNY	F2	
Urbanisation rate	%	F3	
Ratio of resident population to area	%	F4	
Percentage of persons in tertiary education	%	F5	
Digital R&D intensity	%	F6	
Number of employees in the tertiary sector	Person	F7	
Percentage of tertiary sector	%	F8	
Road density	km/km^2^	F9	
Percentage of total exports and imports	%	F10	

**Table 5:** Hyperparameters and performance of different machine learning algorithms.

Models	Optimal Hyperparameters	R^2^
DT	mat_depth: 10, min_samples_splite: 5	0.3877
RF	max_depth: 20, min_samples_splite: 2, n_estimators: 200	0.6806
KNN	n_eighbors: 7, weights: uniform	0.198
SVR	C: 100, gamma: scale, kernel: rbf	0.1884
LightGBM	learning_rate: 0.1, n_estimators: 200, num_leaves: 50	0.7294
CatBoost	depth: 8, iterations: 200, learning_rate: 0.1	0.7283
XGBoost	learning_rate: 0.1, max_depth: 8, n_estimators: 200	0.6974
AdaBoost	learning_rate: 0.1, n_estimators: 50	0.3012
GBDT	learning_rate: 0.1, max_depth: 5, n_estimators: 200	0.6635

## Data Availability

The data and code used in this study can be reasonably requested from the corresponding author.
